# Plant Secondary Metabolites Used for the Treatment of Diseases and Drug Development

**DOI:** 10.3390/biomedicines10030576

**Published:** 2022-03-01

**Authors:** Pavel B. Drašar

**Affiliations:** Department of Chemistry of Natural Compounds, University of Chemistry and Technology, Technicka 5, 166 28 Prague, Czech Republic; pavel.drasar@vscht.cz; Tel.: +420-220-443-698

The importance of natural products in medicine, and in particular, plant secondary metabolites used for the treatment of diseases and drug development, has been obvious for several thousands of years. Thus, this Special Issue of MDPI’s *Biomedicines* has collected the eight top issues from the field as regular full papers, namely, e.g., the investigation of leoligin derivatives as the transcription factor NF-κB, an essential mediator of inflammation NF-κΒ, inhibitory agents. A broad study was made possible using the modular total synthesis method of leoligin, which enabled modifications at two positions, yielding the investigation of the influence of these modifications on the biological activity [1].

Another study showed plant alkaloids inhibiting membrane fusion mediated by calcium and fragments of MERS-CoV and SARS-CoV/SARS-CoV-2 fusion peptides in search of the rationalization of the antiviral actions of plant alkaloids [2].

A further study showed that the oral use of the capsaicinoids (the pungent principles of chilli peppers and prototypical activators of the transient receptor potential of the vanilloid type-1 channel administration) alters the plasma endocannabinoidome and faecal microbiota of reproductive-aged women living with overweight and obesity [3].

An interesting paper described betulinic acid decorated with polar groups and blue-emitting BODIPY dye. This paper described the synthesis of betulinic acid derivatives, their cytotoxicity, cell-cycle analysis, and anti-HIV profiling. The results of this study suggest that betulinic acid has a similar mechanism of inhibition as described in bevirimat [4].

Other topics that were studied include the combined effects of branching and elongation of phytocannabinoids on their bioactivity profile [5], triterpenoid–PEG ribbons targeting selectivity in pharmacological effects [6] showing antimicrobial activity, especially on *Staphylococcus aureus*, *Pseudomonas aeruginosa*, and *Enterococcus faecalis*, and the downregulation of lipogenic gene expression and the attenuation of lipid accumulation through the modulation of the LXRα/SREBP1 pathway in HepG2 cells via tanshinone IIA [7].

A paper in this Special Issue also described the antitumor effects of ursolic acid, a pentacyclic triterpenoid derived from medicinal herbs, through the mediation of the inhibition of STAT3/PD-L1 signalling in non-small cell lung cancer cells [8].

Finally, this Special Issue contains five reviews. One concerns the antitumor activities of curcumin, a main bioactive component of the *Curcuma longa* L. rhizome, and the recent advancements to improve its oral bioavailability that mainly limited its use [9]. A second review shows natural products, alone or in combination with US Food and Drug Administration-approved drugs, used to treat COVID-19, which is a public health emergency of international concern, and lung cancer, a malignant tumour with the highest mortality rate, which has presented significant challenges to both human health and economic development [10]. The third review describes 20-hydroxyecdysone, a polyhydroxylated steroid, and its path from plant extracts to clinical use, mainly showing its therapeutic potential for the treatment of neuromuscular, cardio-metabolic, and respiratory diseases [11]. This review is connected to the fourth one, which names the natural products that “changed society”, as, despite the impressive results achieved within the art of synthetic chemistry, natural products or modified natural products still constitute almost half of the drugs used for the treatment of cancer and diseases such as malaria, onchocerciasis (river blindness), and lymphatic filariasis (caused by parasites) [12]. The fifth review discusses *Cannabis sativa* in terms of interdisciplinary strategies and avenues for medical and commercial progression outside of CBD and THC use [13].
